# Establishing the criterion validity of self-report measures of adherence in hemodialysis through associations with clinical biomarkers: A systematic review and meta-analysis

**DOI:** 10.1371/journal.pone.0276163

**Published:** 2022-10-18

**Authors:** Helena Sousa, Oscar Ribeiro, Elísio Costa, Alan Jay Christensen, Daniela Figueiredo

**Affiliations:** 1 CINTESIS@RISE, Department of Education and Psychology, University of Aveiro, Aveiro, Portugal; 2 Research Unit on Applied Molecular Biosciences (UCIBIO–REQUIMTE), Faculty of Pharmacy and Competence Center on Active and Healthy Ageing (Porto4Ageing), University of Porto, Porto, Portugal; 3 Department of Psychology, East Carolina University, Greenville, North Carolina, United States of America; 4 CINTESIS@RISE, School of Health Sciences, University of Aveiro, Aveiro, Portugal; Medical University of Gdansk, POLAND

## Abstract

Accurate measurement of adherence is crucial to rigorously evaluate interventions aimed at improving this outcome in patients undergoing in-center hemodialysis. Previous research has shown great variability in non-adherence rates between studies, mainly due to the use of different direct (e.g., clinical biomarkers) and indirect (e.g., questionnaires) measures. Although self-reported adherence in hemodialysis has been widely explored, it is still unclear which is the most accurate questionnaire to assess this outcome; therefore, the question of how to optimize adherence measurement in research and clinical practice has emerged as a key issue that needs to be addressed. This systematic review and meta-analysis aimed to explore the criterion validity of self-report measures of adherence in hemodialysis established through the association between test scores and clinical biomarkers (the criterion measure). The protocol was registered in PROSPERO (2021 CRD42021267550). The last search was performed on March 29^th^, 2022, on Web of Science (all databases included), Scopus, CINHAL, APA PsycInfo, and MEDLINE/PubMed. Twenty-nine primary studies were included, and thirty-eight associations were analyzed. The Hunter-Schmidt’s meta-analysis was computed for the associations with more than two studies (*n* = 20). The results showed that six associations were large (16%), 11 were medium (29%) and the remaining were of small strength. The test scores from the End-Stage Renal Disease Adherence Questionnaire (range: 0.212<*r*_c_ <0.319) and the Dialysis Diet and Fluid Non-Adherence Questionnaire (range: 0.206<*r*_c_ <0.359) had medium to large strength associations with interdialytic weight gain, serum phosphorus, and potassium levels, indicating that these questionnaires have reasonable concurrent criterion validity to measure fluid control and adherence to dietary restrictions in patients receiving hemodialysis. The available data did not allow exploring the criterion validity of the test scores in relation to hemodialysis attendance (skipping and/or shortening sessions). These results suggest that the decision to use one questionnaire over another must be made with caution, as researchers need to consider the characteristics of the sample and the objectives of the study. Given that direct and indirect methods have their advantages and disadvantages, the combination of adherence measures in hemodialysis is recommended to accurately assess this complex and multidimensional outcome.

## Introduction

Worldwide, approximately 89% of patients with kidney failure are being treated with hemodialysis; however, treatment success largely depends on patients’ adherence to various health behaviors that are crucial to quality of life and survival [1, 2]. Adherence in hemodialysis has been defined as the extent to which the patient’s behavior corresponds to the medical recommendations agreed with dialysis care professionals [3]. In the context of in-center maintenance hemodialysis, patients need to travel to a dialysis center three to four times a week to receive treatment without missing or shortening sessions, follow strict dietary and fluid restrictions, polypharmacy prescriptions, practice regular exercise, care for the vascular access, and attend follow-up consults [3–5]. Consequently, this renal replacement therapy has been described as one of the most burdensome treatment regimens, and low adherence to all these health behaviors and lifestyle changes is currently considered a major public health problem [6].

However, great variability in non-adherence rates has been reported [7–13]. For instance, the management of dietary restrictions has been pointed out as one of the most challenging behaviors with non-adherence rates ranging from 1.2 to 82.4%, immediately followed by medication intake (1.2 to 81%), and fluid control (3.4 to 74%) [7–12]. Research has also evidenced that non-adherence to in-center hemodialysis sessions varies widely, as 8 to 31% of patients choose to withdraw, withhold, or discontinue treatment [13], whereas 30 to 86% fail to attend dialysis sessions as prescribed [14]. This discrepancy has been mainly attributed to the lack of a gold standard assessment of adherence, as several direct (e.g., biochemical and physiological markers) and indirect (e.g., questionnaires) measures have been used to assess the different domains of adherence in hemodialysis [15].

Regarding direct assessment, studies have relied on clinical biomarkers such as interdialytic weight gain (IDWG), pre-dialysis serum potassium, and/or phosphorous levels as objective measures of fluid and dietary non-adherence, and medication intake [16]. However, the lack of universally accepted cutoff values for each indicator makes comparisons between studies difficult [15, 17]. In turn, self-report measures have been increasingly applied in research and renal care settings, since they are generally inexpensive, flexible, easy to interpret, timesaving, and allow for wide use [18]. The ability to understand patients’ perceptions and explore the reasons for non-adherence is another advantage of indirect measurement [19]. In this context, several questionnaires have been used, namely the End-Stage Renal Disease Adherence Questionnaire (ESRD-AQ), the Dialysis Diet and Fluid Non-Adherence Questionnaire (DDFQ), the Renal Adherence Behavior Questionnaire (RABQ), and other researcher designed/modified instruments [15]; however, their characteristics vary substantially in terms of domain assessed, single or multiple items, and recall period [20]. Furthermore, some lack the necessary validity studies and, therefore, it is still unclear which is the most appropriate to assess self-reported adherence in hemodialysis [9, 11].

To broaden the understanding of the psychometric properties of self-report measures of adherence in hemodialysis, this systematic review and meta-analysis explored their criterion validity established through the association between test scores and clinical biomarkers (the criterion measure). There are two forms of criterion validity: concurrent (an index of the degree to which a test score is related to some criterion measure obtained approximately at the same time, i.e., concurrently) and predictive validity (the degree to which a test score predicts some criterion measure obtained at a future time, i.e., how accurately scores on the test predict some criterion measure) [21, 22]. The use of biochemical data as a criterion measure was based on previous research describing that the criterion validity of self-reported adherence is established through comparison with clinical and biological outcomes, which has been explored in other chronic diseases such as HIV, hypertension, and diabetes [18, 20].

Having this knowledge is of utmost importance, as optimizing the measurement of adherence in hemodialysis is crucial for several reasons. For research, validity is one of the most important psychometric qualities of an instrument, being a synonym for its accuracy [21]. The use of valid measures is also necessary for targeting and rigorously evaluating the growing number of randomized clinical trials that aim to maximize adherence in patients undergoing maintenance hemodialysis [10]. In addition, improving adherence reporting in this population is crucial to support evidence-based public health decision-making, as policymakers often rely on prevalence rates and prediction studies to identify high-risk patients and fund the development of innovative interventions [18].

## Methods

The findings were reported using the 2020 Preferred Reporting Items for Systematic Reviews and Meta-Analysis (PRISMA) statements [23]. The protocol for this study was registered on PROSPERO (2021 CRD42021267550) (see S1 File). The performance of meta-analyses is an addendum to the previously registered protocol. For more information regarding the PRISMA 2020 checklist, see S1 Checklist.

### Eligibility criteria

Following the PICOS structure [24, 25], studies were included based on the following criteria:

- **Participants:** Studies that assessed adults with kidney failure (aged 18 years or older) undergoing in-center hemodialysis. Research reporting the outcomes of patients on home hemodialysis was not considered; studies that focused primarily on other renal replacement therapies, such as peritoneal dialysis or kidney transplantation, were also excluded. This decision was based on the results of previous research suggesting that in-center hemodialysis is the most demanding treatment modality with the highest non-adherence rates [26, 27]. No limits were applied for the length of time on in-center hemodialysis.
- **Exposure:** Studies that exposed patients to self-report measures that aimed to assess one or more domains of adherence in hemodialysis, namely attendance (shortening and/or skipping dialysis sessions), dietary restrictions, fluid control, and medication intake [9, 10, 15]. Questionnaires developed by researchers specifically for their study purpose, visual analog scales, and/or measures without validation data in patients receiving hemodialysis were excluded.
- **Comparator:** Studies assessing clinical biomarkers likely to be affected by the different domains of adherence in hemodialysis, according to the most recently published National Kidney Foundation’s guidelines (NKF) [3, 28, 29].
- **Outcomes:** The criterion validity of self-reported adherence measures was established through the strength of the association between test scores and clinical biomarkers likely to be affected by patients’ adherence [18, 22]; therefore, only studies reporting the results of these associations were considered eligible.
- **Study design:** Only prospective and cross-sectional studies with quantitative data were included.

### Searches

Studies were identified by searching Scopus, Web of Science Core Collection, Current Contents Connect, Derwent Innovations Index, KCI-Korean Journal Database, Russian Science Citation Index, SciELO Citation Index, MEDLINE/PubMed, CINHAL, and APA PsycInfo. The following keywords were used interchangeably: (dialysis OR hemodialysis OR haemodialysis OR end-stage renal disease OR end-stage kidney disease OR renal failure OR renal replacement therapy) AND (adherence OR compliance OR self-management OR self-care) AND (self-report OR questionnaire OR measure OR scale OR instrument) AND (albumin OR potassium OR phosphorus OR phosphate OR interdialytic weight OR IDWG OR Kt/V OR calcium OR sodium OR creatinine OR biomarkers). The search strategy was based on the inclusion criteria, other reviews related to adherence in hemodialysis [26], and the most recently published NKF guidelines [3, 28, 29]. Limits were applied to the English language and research with adults. Other reviews, protocols, practice guidelines, or conference abstracts were not considered. Studies published before 2000 were also excluded; this time limit has been established in accordance with the release of the first NFK Dialysis Outcome Quality Initiative clinical practice guidelines for hemodialysis adequacy and nutritional management in chronic kidney disease [30, 31]. The reference lists of the identified studies and other reviews were hand-searched to ensure that all-important studies were included. The electronic search was performed between July 1^st^ and 14^th^, 2021, and updated on March 29^th^, 2022. For more information about the search process, see S1 Table.

### Study selection and data collection

Studies were extracted from the databases and exported to Rayyan, a software designed to facilitate the study selection in reviews (https://www.rayyan.ai/). After manually removing duplicates, the unique studies were selected over three steps: (i) studies were labeled as included, excluded, or unclear, based on title and abstract; (ii) those studies labeled as unclear or as included were retrieved; and (iii) the full text was analyzed. Two authors [HS and DF] independently performed the eligibility assessment with an inter-rater agreement of 88.2%. Discrepancies were resolved by discussion and consensus, and by consulting with a third author [OR]. The following data were retrieved: study characteristics (date, design, and sample size), sample characteristics (age, sex, country, and length of time on dialysis), self-report measures of adherence and results, biomarkers likely to be affected by adherence and results, statistics used to explore the association between self-report measures and biomarkers, and the major results for this association.

### Critical appraisal

The quality of the included studies was critically appraised using the Joanna Briggs Institute (JBI) Critical Appraisal Checklists for Analytical Cross-Sectional Studies. This checklist aims to assess the methodological quality of a study regarding the possibility of bias in its design, conduct, and analysis. Each item on these checklists is appraised as “yes”, “no”, “unclear” or “not applicable”. Two authors [HS and EC] conducted the appraisal with an IR agreement of 87.5%. Discrepancies were solved by discussion and consensus, and by consulting with a third author [DF].

### Strategy for data synthesis

The validity coefficient was used as an effect size measure [22]. This refers to a correlation coefficient that provides a measure of the strength of the relationship between the test scores (i.e., self-report measures of adherence) and results of the other measure of interest (i.e., biomarkers) [22].

Hunter-Schmidt’s psychometric meta-analyses [32] using the random-effects model were computed for associations with more than two studies (*k* ≥ 2). The ‘bare-bone’ mean of *r* (*r*_c_) corrected for sampling error was calculated by weighting each correlation coefficient with the respective sample size. Values of 0.12, 0.20, and 0.32 were interpreted as small, medium, and large [33]. Heterogeneity among studies was evaluated using both the Chi-squared test and the I-squared statistic. A value of < 0.10 was considered to indicate statistically significant heterogeneity between studies [34]. An I-squared value of 25% represents a small degree of heterogeneity, 50% a moderate degree, and 75% a large degree of heterogeneity [34]. All the analyses were performed using R version 4.2.1 (R Foundation for Statistical Computing, Vienna, Austria). The “meta” package with the function “metafor” was used to perform the meta-analyses.

## Results

### Study selection

**Fig 1:**
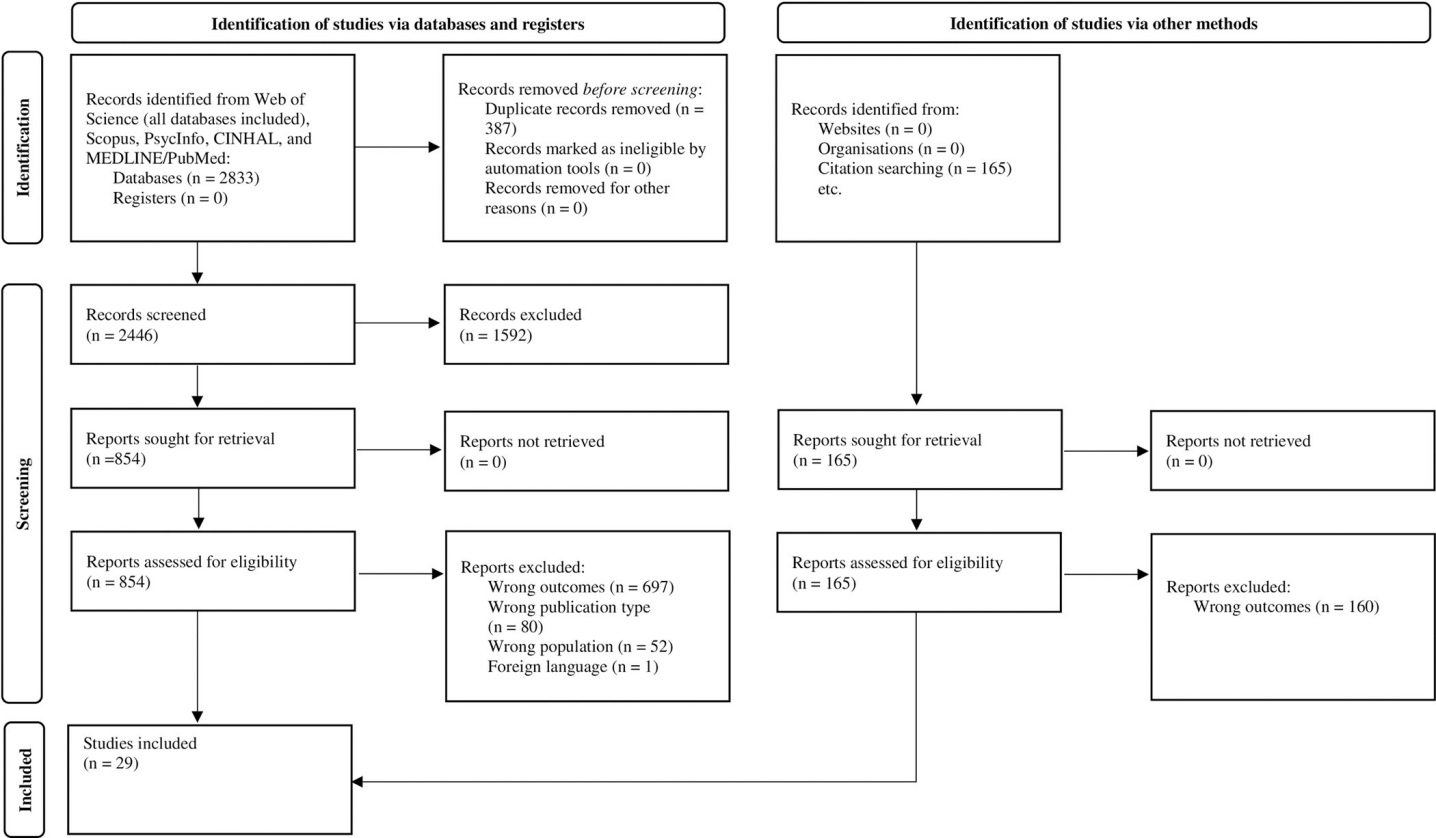
PRISMA 2020 flow diagram.

Twenty-nine primary studies met the inclusion criteria for this systematic review and meta-analysis. Fig 1 presents the different stages of study selection including reasons for exclusion.

### Study characteristics

The studies were published from 2001 to 2021. Seventeen (59%) were published after (and including) 2015. All studies had a cross-sectional design. Pearson’s product-moment correlation (*n* = 16, 55%) and Spearman’s rank correlation (*n* = 7, 24%) were the most frequently used coefficients to explore the association between test scores and clinical biomarkers. Table 1 shows the characteristics of the included studies and major results.

**Table 1:** Characteristics of the primary studies (*n* = 29) and major results.

Reference	Sample [age; sex; race/ethnicity]	Length of time on dialysis (month cut-off and mean/SD in months)	Self-reported non-adherence (%)	Clinical biomarkers	Statistics used to explore the criterion validity	Results of the association between test scores and clinical biomarkers
**Ahrari et al., 2014 (Iran)** [7]	237 patients [46.1±15.4y; 57.5% men; NR]	> 3	DDFQ Degree of NAdh to diet: 91.8% Degree of NAdh to fluids: 86.3%	IDWG: 1.49±0.71 SPO: 6.37±1.7 SK: 4.76±0.75 ALB: 4.09±0.36	Spearman Rho	Degree of NAdh to diet ⬄ IDWG (*r*_*s*_ = 0.518*), SPO (*r*_*s*_ = 0.651*), SK (*r*_*s*_ = 0.324*), and ALB (*r*_*s*_ = 0.319*) Degree of NAdh to fluids ⬄ IDWG (*r*_*s*_ = 0. 575*), SPO (*r*_*s*_ = 0.537*), SK (*r*_*s*_ = 0.277*), and ALB (*r*_*s*_ = 0.338*)
**Amado et al., 2015 (Portugal)** [35]	122 patients [64.1±15.3y; 58.7% women; NR]	235.5±8.4	MMAS-8 Adh to antihypertensives: 10.7%	BP diastolic: 65.9±13.7 BP systolic: 140.1±16.9	Pearson R	Adh to antihypertensives ⬄ BP diastolic (*r* = 0.286*), and BP systolic (*r* = NR, ns)
**Antony et al., 2020 (India)** [36]	121 patients [62.1±11.4y; 77% men; NR]	<12: 14.1%; 13–60: 62.8%; 61–120: 18.2%; >120: 4.96%	ESRD-AQ NAdh to diet: 36.7% NAdh to fluids: 39.7% NAdh to PB: 32.3% NAdh to HD: NR	IDWG: 2.95±1.19 SPO: 5.67±1.70 SK: 4.96±1.01	Pearson R	NAdh to diet ⬄ IDWG (*r* = 0.135), SPO (*r* = 0.407*), SK (*r* = 0.335*) NAdh to fluids ⬄ IDWG (*r* = 0.410*), SPO (0.411), SK (*r* = 0.02) NAdh to PB ⬄ SPO (*r* = 0.306*) NAdh to HD ⬄ NR
**Anuja et al., 2020 (India)** [37]	96 patients [62.1±11.4y; 66.7% men; NR]	> 6	ESRD-AQ NAdh to diet: 40.6% NAdh to fluids: 62.5% NAdh to meds: 28.6% NAdh to HD (shortening sessions): 7.5% NAdh to HD (skipping sessions): 8.3%	IDWG: values NR	Logistic Regression	Overall NAdh ⬄ >2kgs IDWG (*OR* = 4.7, 95% *CI* = 0.6–2.7)
**Chan et al., 2012 (Malaysia)** [38]	188 patients [58.2±10.5y; 51.1% women; NR]	63.2±39.3	DDFQ Degree of NAdh to diet: 63.8% Degree of NAdh to fluids: 51.6%	IDWG: 2.8±0.7 SPO and SK: values NR	Pearson R	Degree of NAdh to diet ⬄ IDWG (*r* = 0.174, ns), and SPO + SK (*r* = 0.25) Degree of NAdh to fluids ⬄ IDWG (*r* = 0.342*), and SPO + SK (NR)
**Chao et al., 2016 (Taiwan)** [39]	51 patients [68±11.8y; 57% women; NR]	40.8±33.6	MMAS-8 Adh to medication (including PB): 39%	SPO: 5.3±1.5 SK: 4.70±0.7 ALB: 3.8±0.3 Ca: 8.9±0.8 Ca x SPO: 47.3±14 Na: 135±3.9 Glucose: 107±36 Cholesterol: 158±40 BUN: 80.8±19.9 Creatinine: 10.6±2.5 Hgl: 9.7±1.3 Ferritin: 647±784 Kt/V: 1.6±0.2 URR: 74.7±5.90	Pearson R	Adh to meds ⬄ Kt/V (*r* = 0.29*). Remaining associations: reported as ns
**Chen et al., 2021 (China)** [40]	226 [55.4±14.7; 67% men; NR]	51±47.7	FAS NAdh to fluids: NR	IDWG: 4.20±1.52	Structural Equation Modeling	NAdh to fluids ⬄IDWG (*β* = 0.40*, 95% *CI* NR)
**Daniels et al., 2018 (USA)** [41]	120 patients [59.9±15.4y; 53.3% women; 100% Non-White]	66±60	ESRD-AQ NAdh to diet: 75.8% NAdh to fluids: 63% NAdh to meds: 20.8% NAdh to HD (shortening and/or skipping sessions): 19.2%	IDWG: 2.82 SPO: 5.27 SK: 4.8	Pearson R	NAdh to diet ⬄ SPO, and SK (ns, but Pearson *R* NR) NAdh to fluids ⬄ IDWG (p < .005, but Pearson *R* NR) NAdh to meds ⬄ NR NAdh to HD ⬄ NR
**Efe et al. 2015 (USA)** [8]	121 patients [21-35y: 13%; 36-50y: 22%; 61-65y: 65%; 62% men; NR]	> 6	DDFQ Degree of NAdh to diet: 98.3% Degree of NAdh to fluids: 95%	SPO, SK, Ca, Na, ALB, and Hgl: values NR	Spearman Rho	Degree of NAdh to diet ⬄ SPO (*r*_*s*_ = 0.18*), SK (*r*_*s*_ = 0.17), Ca (*r*_*s*_ = 0.05), Na (*r*_*s*_ = 0.10), ALB (*r*_*s*_ = 0.05), and Hgl (*r*_*s*_ = -0.08) Degree of NAdh to fluids ⬄ SPO (*r*_*s*_ = 0.28*), SK (*r*_*s*_ = 0.11), Ca (*r*_*s*_ = 0.12), Na (*r*_*s*_ = 0.05), ALB (*r*_*s*_ = -0.12), and Hgl (*r*_*s*_ = 0.12)
**Fincham et al., 2008 (South Africa)** [42]	62 patients [40.3±9.4y; 58.1% women; 93.5% Non-White]	91.1±87.1	RABQ Overall NAdh: NR	IDWG, SPO, and SK: values NR	Pearson R	Overall NAdh ⬄ IDWG (*r* = 0.05), SPO (*r* = 0.02), and SK (*r* = -0.15)
**Ghimire et al., 2016 (Australia)** [43]	53 patients [67.9±11.5y; 58.5% men; NR]	44.4±40.8	MMAS-4 Adh to PB: 56.7%	SPO: 5.30±1.50	Pearson R	Adh to PB ⬄ SPO (*r* = 0.43*)
**Joson et al., 2016 (USA)** [44]	79 patients [62.6±15y; 53.2% men; 92% Non-White]	58.9±41.5	MMAS-8 Adh to PB: NR	SPO: 4.44±0.76	Pearson R	Adh to PB ⬄ SPO (*r* = 0.18)
**Kara et al., 2007 (Turkey)** [45]	160 patients [57±14.5y; 57.5% men; NR]	47.3±39.2	DDFQ Degree of NAdh to diet: 28.1% Degree of NAdh to fluids: 41.2%	IDGW: 2.4±0.8 SPO: 4.9±0.9 SK: 5.60±0.6 ALB: 3.7± 0.4	Pearson R	Degree of NAdh to diet ⬄ IDGW (*r* = 0.31*), SPO (*r* = 0.27*), SK (*r* = 0.39*), and ALB (*r* = -0.16*) Degree of NAdh to fluids ⬄ IDGW (*r* = 0.71*), SPO (*r* = 0.14), SK (*r* = 0.21*), and ALB (*r* = 0.14*)
**Katalinić et al., 2017 (Croatia. Montenegro. Bosnia and Herzegovina)** [46]	417 patients [63.8y; 55.1% men; NR]	68.6	MMAS-8 Adh to PB: NR	SPO: 4.9±0.9 SK: 5.60±0.6 ALB: 3.7±0.4 Kt/V: 1.31 iPHT: 41.4	Spearman Rho	Adh to PB ⬄ SPO (*r*_*s*_ = 0.192*), and PTHi (*r*_*s*_ = 0.074)
**Kauric-Klein et al., 2013 (USA)** [47]	118 patients [59.7±15.9y; 51% men; 88% Non-White]	>6 m	MMAS-4 Adh to antihypertensives: NR	BP diastolic: 87.4±10.2 BP systolic: 163.6±12.4	Pearson R	Adh to antihypertensives ⬄ BP systolic (*r* = 0.20*), and BP diastolic (*r* = 0.26*)
**Khalil et al., 2013 (Jordan)** [48]	190 patients [49 ±14.9y; 54% men; NR]	70.8±63.6	DDFQ Degree of NAdh to diet: 71% Degree of NAdh to fluids: 77%	IDGW: 3.3±1.2 SPO: 4.20±1.50 SK: 4.80±0.80	Spearman Rho	Degree of NAdh to diet ⬄ NR Degree of NAdh to fluids ⬄ IDWG (*r*_*s*_ = 0.181*)
**Kim et al., 2010 (USA)** [9]	151 patients [51.9 ±15.6y; 57.6% men; 97.3% Non-White]	51.3±49.7	ESRD-AQ NAdh to diet: 31.8% NAdh to fluids: 20.5% NAdh to PB: 31.8% NAdh to HD (shortening sessions): 15.9% NAdh to HD (skipping sessions): 9.3%	IDWG: 2.75±1.01 SPO: 5.55±1.69 SK: 4.98±0.71 Kt/V: 1.69±0.33	Pearson R	NAdh to diet ⬄ IDWG (*r* = 0.141), SPO (*r* = 0.056), SK (*r* = 0.051), and Kt/V (*r* = 0.069) NAdh to fluids ⬄ IDWG (*r* = 0.205*), SPO (*r* = 0.042), SK (*r* = 0.041), and Kt/V (*r* = -0.010). NAdh to PB ⬄ SPO (*r* = 0.272*) NAdh to HD ⬄ NR
**Kugler et al., 2005 (Belgium and Germany)** [49]	484 patients [67y. 52.9% men; NR]	47	DDFQ Degree of NAdh to diet: 81.4% Degree of NAdh to fluids: 74.6%	IDWG: 2.15±1.06 SPO: 4.87±4.60 SK: 4.99±0.81 ALB: 4.51±2.23	Spearman Rho	Degree of NAdh to diet ⬄ IDWG (*r*_*s*_ = 0.258**), SPO (*r*_*s*_ = 0.072*), SK (*r*_*s*_ = 0.109*), ALB (*r*_*s*_ = 0.203*) Degree of NAdh to fluids ⬄IDWG (*r*_*s*_ = 0.351*), SPO (*r*_*s*_ = 0.099*), SK (*r*_*s*_ = 0.092*), ALB (*r*_*s*_ = 0.180*)
**Lim et al., 2020 (Malaysia)** [50]	218 patients [54.8±12.8y; 53.2% men; 100% Non-White]	67.2±54.3	ESRD-AQ NAdh to diet: 65% NAdh to fluids: NR NAdh to PB: NR NAdh to HD: NR	SPO and SK: values NR	Pearson R	NAdh to diet ⬄ SPO (*r* = 0.261*), SK (*r* = 0.184*)
**Mellon et al., 2013 (Ireland)** [51]	50 patients [57±15.9y; 60% men; NR]	48±37.2	RABQ NAdh to diet and fluids (not discriminated): NR	IDWG: 2.27±1.07 SPO: 5.00±1.50 SK: 5.17±0.54	Pearson R	Overall NAdh ⬄ IDWG (*r* = 0.253), SPO (*r* = 0.261), SK (*r* = 0.153)
**Mollaoğlu et al., 2015 (Turkey)** [52]	186 patients [<30y: 10.8%; 31-60y: 63.5; >61y: 25.8%; 53.2% men; NR]	>3 m	DDFQ Degree of NAdh to diet: 66.7% Degree of NAdh to fluids: 68.8%	IDWG: 2.3±0.74	Logistic Regression	Degree of NAdh to diet ⬄ IDWG (*OR* = 1.45*, 95% *CI* 1.12–1.80) Degree of NAdh to fluids ⬄ IDWG (*OR* = 2.61*, 95% *CI* 1.36–6.20)
**Naalweh et al., 2017 (Palestine)** [53]	220 patients [56.8±14.5y; 52.8% men; NR]	48.2±44.4	ESRD-AQ NAdh to diet: NR NAdh to fluids: NR NAdh to PB: NR NAdh to HD: NR	IDWG: 3.10±1.63 SPO: 4.74±0.59 SK: 4.95±0.74	Spearman Rho	NAdh to diet ⬄ IDWG (*r*_*s*_ = 0.270*), SPO (*r*_*s*_ = 0.108), SK (*r*_*s*_ = 0.281*) NAdh to fluids ⬄ IDWG (*r*_*s*_ = 0.432*), SPO (*r*_*s*_ = 0.081), SK (*r*_*s*_ = 0.128) NAdh to PB ⬄ SPO (*r*_*s*_ = 0.071) NAdh to HD ⬄ NR
**Ok et al., 2019 (Turkey)** [54]	90 patients [53.7±12.7y; 54.4% women; NR]	63.5±49.4	ESRD-AQ Overall NAdh: NR	IDWG: 1.42±0.39 SPO: 5.04±0.99 SK: 5.15±0.29 ALB: 4.26±0.33	Spearman Rho	Overall NAdh ⬄ IDWG (*r*_*s*_ = 0.533*), SPO (*r*_*s*_ = 0.237*), SK (*r*_*s*_ = 0.168), and ALB (*r*_*s*_ = -0.04)
**Poveda et al., 2016 (Portugal)** [11]	185 patients [66.4±14.3y; 50.3% women; NR]	62.5±58	ESRD-AQ NAdh to diet: 56.2% NAdh to fluids: 50.3% NAdh to meds: 15.7% NAdh to HD (shortening and/or skipping sessions): 6.5%	IDWG: 2.1±0.8 SPO: 4.25±1.2 SK: 5.15±0.8 ALB: 4.0±0.3 Hgl: 11.5±1.6 Kt/V: 1.7±0.3 BP diastolic: 63.8±12.9 BP systolic: 134.9±20.7	Pearson R	NAdh to fluids ⬄ IDWG (*r* = 0.227*)
**Umeukeje et al., 2015 (USA)** [55]	100 patients [51±15.2; 53% men; 72% Non-White]	NR	MMAS-8 NAdh to PB: NR	SPO: 5.8±1.6	Pearson R	NAdh to PB ⬄SPO (*r* = 0.40*)
**Umeukeje et al., 2016 (USA)** [56]	296 patients [55±15.3; 51% women; 63% Non-White]	NR	MMAS-8 NAdh to PB: NR	SPO: 5.5±1.6	Linear Regression Analysis	NAdh to PB ⬄SPO (*β* = 0.15*, 95% *CI* -0.23 − -0.07)
**Vlaminck et al., 2001 (Belgium)** [57]	564 patients [65.9±12.5y; 50.1% women; NR]	44.9±48.7	DDFQ Degree of NAdh to diet: 81.4% Degree of NAdh to fluids: 72%	IDWG: 2.07±1.00 SPO: 4.97±1.67 SK: 4.94±0.72 ALB: 3.82±0.46	Kendall’s Tau	Degree of NAdh to diet ⬄ IDWG (*τ* = 0.197*), SPO (*τ* = 0.108*), SK (τ = 0.084*), and ALB (*τ* = 0.178*) Degree of NAdh to fluids ⬄ IDWG (*τ* = 0.242*)
**Wileman et al., 2014 (UK)** [12]	112 patients [60.5±16.9y; 61.6% men; 81.3% White]	26.4±15.6	MARS Adh to PB: NR	SPO: 6.20±1.00	Pearson R	Adh to PB ⬄ SPO (*r* = 0.42*)
**Wileman et al., 2011 (UK)** [58]	76 patients [63.1±15.4y; 60.5% men; 84.2% White]	64.8±69.6	MMAS-4 NAdh to PB: 14.5%	SPO: 5.70±1.50	Pearson R	NAdh to PB ⬄ SPO (*r* = 0.35*)

Abbreviations or symbols used in the table: ALB = Albumin; β = Beta; BP = Blood Pressure; BUN = Blood Urea Nitrogen; Ca = Calcium; CI = Confidence Interval; DDFQ = Dialysis Diet and Fluid Non-Adherence Questionnaire; ESRD-AQ = End-Stage Renal Disease Adherence Questionnaire; FAS = Fluid Adherence Subscale of the Hemodialysis Patients Therapy Adherence Scale; HD = Hemodialysis; Hgl = Hemoglobin; IDWG = Interdialytic Weight Gain; iPHT = Intact Parathyroid Hormone; MARS = Medication Adherence Rating Scale; MMAS = Morisky Medication Adherence Scale; Na = Sodium; NAdh = Non-adherence; NR = Not Reported; Ns = Not Statistically significant; OR = Odds Ratio; PB = Phosphate Binders; RABQ = Renal Adherence Behavior Questionnaire; r_s_ = Sperman Rho; SD = Standard Deviation; SK = Potassium; SPO = Phosphorus; URR = Urea Reduction Rate.

IDWG is measured in kilograms; SK, SPO, Ca, Glucose, Cholesterol, Creatinine in mg/dl; ALB in g/dl; PB in mmHg; iPHT and Ferritin in ng/l; Sodium in mmol/l.; URR in %.

* Statistically significant association was set at a p-value of <0.05.

Information on the length of time on hemodialysis was obtained from the primary studies (mean and standard deviations were reported whenever available).

### Critical appraisal

All studies clearly defined the inclusion criteria and described the population in detail. The questionnaires used to measure adherence were valid and reliable; however, only fourteen studies (48%) reported reliability results using the study sample. Internal consistency was mostly assessed with Cronbach’s alpha [7, 8, 12, 38, 42, 45, 48, 49, 55, 58] while studies using the ESRD-AQ calculated the inter-class and/or the item-total scale correlation coefficient [9, 11, 41, 54].

Since the type of hemodialysis (conventional or on-line hemodiafiltration), the number of dialysis sessions (per week), the etiology of kidney failure, the type of vascular access, and the level of residual renal function can confound the results of clinical biomarkers [59–61], these data were considered in the critical appraisal of each study. In this sense, only two studies reported that patients were receiving on-line hemodiafiltration [11, 35] while the remaining did not present data regarding the type of hemodialysis. Four studies [11, 35, 40, 54] stated that patients received treatment three times a week for four hours per session, while in two studies [36, 53] most patients were dialyzed twice a week. Only five studies (17%) identified kidney failure etiology, reporting that diabetes was the most common cause, followed by hypertension and glomerulonephritis [9, 11, 38, 39, 58]. Only one study [35] presented information on vascular access, describing that most patients underwent treatment through an arteriovenous fistula. Regarding residual renal function, only four studies (14%) described these data and two of them found that renal urea clearance (KrU) was significantly associated with non-adherence [46, 48].

Lastly, four studies (14%) did not provide information on how/when clinical biomarkers were collected [39, 41, 45, 49]. It is important to emphasize that there was great variability in this regard, which may introduce bias, as some studies (*n* = 6, 21%) stated that data collection took place in the same week as the self-report measure was applied, while others averaged the clinical results of the last weeks before the questionnaire administration (*n* = 7, 24%). For more information on the critical appraisal of all studies see S2 Table; for a detailed description of confounders, see S3 Table.

### Participants’ characteristics

This study comprised a total of 5093 patients with kidney failure (*M* = 176, *SD* = 126, range: 50–564) underdoing in-center hemodialysis for an average of 64.8 months (*SD* = 41.4, range: 40.3–66.4). Six studies [7, 8, 30, 31, 40, 41, 46] reported categorical data, suggesting that patients with more than three months on dialysis were included in their analysis. Most participants were men (54%), with an average of 58.5 years old (*SD* = 6.91, range: 40.3–66.4), and recruited from 19 different countries. Only 10 primary studies reported patients’ race/ethnicity [9, 12, 41, 42, 44, 47, 50, 55, 56, 58]; most participants in these studies were non-White (Table 1).

### Self-reported adherence

Adherence to dietary restrictions (Table 1) and fluid control were equally the most evaluated outcomes (*n* = 15, 52%), followed by medication intake, especially phosphate binders (*n* = 9, 31%). Only four studies (17%) reported patients’ attendance to in-center hemodialysis sessions [7, 11, 37, 41]. Six different self-report measures were applied. The ESRD-AQ (*n* = 8, 28%) and the DDFQ (*n* = 8, 28%) were the most frequently used to assess dietary and fluid restrictions, while the Morisky Medication Adherence Scale (MMAS-4 and MMAS-8; *n* = 9, 21%) was the most applied to assess medication intake. Available data suggested that an average of 63.5% (*SD* = 22.2, range: 31.8–98.3) of patients were non-adherent to dietary recommendations, 61.7% to fluid restrictions (*SD* = 20.7, range: 20.5–86.3), 27.8% to medication intake (*SD* = 14.4, range: 10.7–56.7), and 11.6% (*SD* = 5.71, range: 8–19.2) to in-center hemodialysis sessions (skipping and/or shortening sessions). For a summary of each self-reported adherence measure analyzed in this study, see S4 Table.

### Clinical biomarkers of treatment adherence

Eighteen different biomarkers were used to assess adherence. Serum phosphorus levels (SPO; *n* = 16, 55%), serum potassium levels (SK; *n* = 14, 48%), IDWG (*n* = 14, 48%), serum albumin levels (ALB; *n* = 6, 21%), Kt/V (*n* = 4, 14%), and blood pressure (BP diastolic and systolic; *n* = 3, 10%) were the most frequently collected parameters. SPO, SK, and ALB were used to assess adherence to diet, while SPO also measured phosphate binder’s intake. IDWG was mainly used to assess fluid control, while BP was collected to evaluate adherence to antihypertensives. Available data (Table 1) suggested that the mean SPO value was 5.20 mg/dl (*SD* = 0.59, range: 4.2–6.37), SK was 5.04 mg/dl (*SD* = 0.28, range: 4 .70–5.60), and ALB was 3.99 g/dl (*SD* = 0.26, range: 3.70–4.51). Patients also had an average IDWG of 2.54 kilograms (*SD* = 0.41, range: 1.42–4.20) with an average Kt/V of 1.56 (*SD* = 0.18, range: 1.31–1.7). Regarding physiological data, the mean value of systolic BP was 146 mmHg (*SD* = 14.9, range: 135–163), while the mean diastolic BP was 72.4 mmHg (*SD* = 72.4, range: 63.8–87.4).

### Criterion validity

In the primary studies, the application of the questionnaires was completed at approximately the same time as the collection of laboratory results (for details, see S3 Table); therefore, it was not possible to report data on the predictive value of self-report measures (predictive validity) and only their concurrent criterion validity was analyzed (Table 2).

**Table 2:** Results of meta-analyses for concurrent criterion-related validity correlation coefficients between test scores and clinical biomarkers of adherence in hemodialysis.

Adherence domain	Self-report measure	Clinical biomarkers	Evidence base	*r*_c_ [*se*; 95% *CI*]	*Q* statistic [*p*-value]	*I*^*2*^ [%]	Concurrent criterion validity⬄ (based on the strength of the association)
**Dietary restrictions**	ESRD-AQ	IDWG	3 studies of 492 patients	0.197 [0.043; 0.112–0.282]*	2.27 [0.321]	0	Small
		SPO	4 studies of 710 patients	0.195 [0.063; 0.071–0.319]*	11.8 [0.008]**	66	Small
		SK	4 studies of 710 patients	0.212 [0.052; 0.113–0.309]*	7.58 [0.055]**	47	Medium
	DDFQ	IDWG	5 studies of 1633 patients	0.271 [0.054; 0.165–0.375]*	22.8 [<0.001]**	77	Medium
		SPO	6 studies of 1754 patients	0.206 [0.088; 0.034–0.378]*	67.5 [<0.001]**	91	Medium
		SK	6 studies of 1754 patients	0.175 [0.049; 0.079–0.271]*	21.7 [<0.001]**	71	Small
		ALB	5 studies of 1566 patients	0.195 [0.031; 0.136–0.255]*	7.07 [0.132]	28	Small
		Ca	1 study of 121 patients	0.05	–	–	Small
		Na	1 study of 121 patients	0.10	–	–	Small
		Hgl	1 study of 121 patients	0.08	–	–	Small
**Fluid control**	ESRD-AQ	IDWG	4 studies of 677 patients	0.319 [0.051; 0.218–0.419]*	8.51 [0.036]**	53	Large
		SPO	3 studies of 492 patients	0.151 [0.089: -0.023–0.324]	11.5 [0.003]**	73	Small
		SK	3 studies of 272 patients	0.075 [0.045; -0.013–0.163]	1.16 [0.559]	0	Small
		Kt/V	1 study of 151 patients	0.01	–		Small
	DDFQ	IDWG	6 studies of 1823 patients	0.359 [0.071; 0.219–0.499]*	58.5 [<0.001]**	89	Large
		SPO	4 studies of 1002 patients	0.227 [0.103; 0.024–0.429]*	36.9 [<0.001]**	88	Medium
		SK	4 studies of 1002 patients	0.156 [0.042; 0.073–0.238]*	6.59 [0.086]**	37	Small
		ALB	4 studies of 1002 patients	0.204 [0.041; 0.123–0.284]*	6.55 [0.088]**	36	Medium
		Ca	1 study of 237 patients	0.12	–	–	Small
		Na	1 study of 237 patients	0.05	–	–	Small
		Hgl	1 study of 121 patients	0.12	–	–	Small
	FAS	IDWG	1 study of 226 patients	0.40	–	–	Large
**Phosphate binders’ intake**	ESRD-AQ	SPO	3 studies of 492 patients	0.191 [0.064; 0.065–0.315]	6.22 [0.045]**	51	Small
	MMAS-4	SPO	2 studies of 129 patients	0.383 [0.076; 0.234–0.531]	0.269 [0.603]	0	Large
	MMAS-8	SPO	4 studies of 892 patients	0.20 [0.038; 0.126–0.275]	5.16 [0.161]	20	Medium
		iPHT	1 study of 417 patients	0.074	–	–	Small
	MARS	SPO	1 study of 112 patients	0.421	–	–	Large
**Antihypertensives’ intake**	MMAS-4	BP diastolic	1 study of 118 patients	0.26	–	–	Medium
		BP systolic	1 study of 118 patients	0.20	–	–	Medium
	MMAS-8	BP diastolic	1 study of 122 patients	0.286	–	–	Medium
		Kt/V	1 study of 51 patients	0.29	–	–	Medium
**Overall non-adherence**	ESRD-AQ	IDWG	1 study of 90 patients	0.533	–	–	Large
		SPO	1 study of 90 patients	0.237	–	–	Medium
		SK	1 study of 90 patients	0.168	–	–	Small
		ALB	1 study of 90 patients	0.04	–	–	Small
	RABQ	IDWG	2 study of 112 patients	0.141 [0.094; -0.043–0.324]	1.17 [0.281]	0	Small
		SPO	2 study of 112 patients	0.128 [0.094; -0.056–0.311]	1.63 [0.202]	0	Small
		SK	2 study of 112 patients	0.151 [0.093; -0.033–0.333]	0.00 [1.00]	0	Small

Abbreviations or symbols used in the table: ALB = Albumin; BP = Blood Pressure; BUN = Blood Urea Nitrogen; Ca = Calcium; CI = Confidence Interval; DDFQ = Dialysis Diet and Fluid Non-Adherence Questionnaire; ESRD-AQ = End-Stage Renal Disease Adherence Questionnaire; Hgl = Hemoglobin; IDWG = Inter-Dialytic Weight Gain; *I*^2^ = I-squared statistic to measure heterogeneity; iPHT = Intact Parathyroid Hormone; MARS = Medication Adherence Rating Scale; MMAS = Morisky Medication Adherence Scale; Na = Sodium; RABQ = Renal Adherence Behavior Questionnaire; *r*_c_ = weighted mean of *r* corrected for sampling error; *se* = standard error; SK = Potassium; SPO = Phosphorus; URR = Urea Reduction Rate.

⬄ Values of 0.12, 0.20, and 0.32 were interpreted as small, medium, and large [33].

* Statistical significant associations were set at *p*<0.05.

** Statistical significant heterogeneity was set at *p*<0.10 for the *Q* statistic.

The ‘bare-bone’ mean of correlation coefficients (*r*_c_) corrected for sampling error was calculated by weighting each *r* with the respective sample size. This statistic was computed for all outcomes with *k* ≥ 2.

The remaining associations (i.e., *k* = 1) refer to the statistics presented for each outcome which were reported in Table 1.

Thirty-eight associations between self-report measures and clinical biomarkers likely to be affected by patients’ adherence were analyzed. Individual meta-analyses were also performed for associations with more than two primary studies (*n* = 20). These refer to dietary restrictions, fluid control, polypharmacy intake, and overall non-adherence; however, the available data did not allow exploring the criterion validity of the test scores in relation to hemodialysis attendance.

In general, six associations were large (16%), 11 were medium (29%) and the rest were of small strength. A large statistically significant and moderately heterogeneous association was found between the ESRD-AQ and IDWG for fluid control (*r*_c_ = 0.319; 95% *CI* = 0.218–0.419; *I*^2^ = 53%; *k* = 4; *n* = 677) [9, 11, 36, 53]. For the same outcome and biomarker, large statistically significant and heterogeneous associations were found with the DDFQ (*r*_c_ = 0.359; 95% *CI* = 0.219–0.499; *I*^2^ = 89%; *k* = 6; *n* = 1823) [7, 38, 45, 48, 49, 57]. Regarding dietary restrictions, both the ESRD-AQ (*r*_c_ = 0.212 with SK; 95% *CI* = 0.113–0.309; *I*^2^ = 47%; *k* = 4; *n* = 710) [9, 36, 50, 53] and the DDFQ (*r*_c_ = 0.206 with SPO; 95% *CI* = 0.034–0.378; *I*^2^ = 91%; *k* = 6; *n* = 1754) [7, 8, 38, 45, 49, 57] showed medium-strength statistically significant associations with clinically relevant biomarkers of this type of adherence. To measure overall non-adherence, the results showed a medium association between the ESRD-AQ scores and SPO levels (*r*_*s*_ = 0.237; *k* = 1; *n* = 90) [54] and a large association with IDWG values (*r*_*s*_ = 0.533; *k* = 1; *n* = 90) [54]. Compared with other patient-reported outcome measures, the MMAS had the most medium to large-strength associations with medication adherence outcomes (SPO: 0.20 < *r*_c_ < 0.383; BP: 0.20 < *r* < 0.286) [35, 43, 44, 46, 47, 55, 58].

## Discussion

This systematic review with Hunter-Schmidt’s psychometric meta-analyses reports the criterion validity of self-report measures of adherence in hemodialysis, through the strength of the association between test scores and clinical biomarkers (the criterion measure).

Overall, the results showed that both the ESRD-AQ and the DDFQ have reasonable concurrent criterion validity with biomarkers that measure adherence to fluid and dietary restrictions; therefore, the choice of the most appropriate tool may depend on the objectives of each study and/or characteristics of the sample. On the one hand, the ESRD-AQ allows for a more comprehensive assessment of treatment adherence, as it measures different domains (hemodialysis attendance, medication intake, fluid, and dietary restrictions) but also reports on reasons for non-adherence, and patients’ perceptions and understanding of medical recommendations [9, 11, 62]. However, the readability of this instrument, as well as the patients’ ability to understand each question, has been poorly evaluated in validation studies [9]. On the other hand, the DDFQ may be more intelligible due to its lower complexity [9, 57] and therefore more appropriate if the study sample has low literacy levels. Nonetheless, some concerns have been raised due to its modest psychometric qualities (moderate construct validity) and over-simplified design, as it consists of only four questions that assess frequency and degree of adherence to fluid and dietary restrictions [9, 57]. In comparison, the ESRD-AQ has good construct and content validity and excellent test-retest reliability [9], which evidences that this tool is valuable for researchers and clinicians working with patients with kidney failure requiring maintenance in-center hemodialysis [9, 11] (see S4 Table).

In addition, the results showed that IDWG had the strongest correlations with self-reported fluid control and overall non-adherence, suggesting that this indicator may be the most sensitive marker to measure these outcomes in this population. Considering the results of each primary study, stronger correlations were found when IDWG values were averaged six to 12 sessions before the questionnaire application [52–54] (see S3 Table), which allows us to hypothesize that this may be a reliable way to use this parameter to measure adherence in future research. However, studies need to control for residual urinary output and extrarenal fluid losses to increase the reliability of this measure [48, 49], which only occurred in four of the 29 primary studies included in this review.

Finally, it is important to note that the strength of associations between self-reported adherence and clinical biomarkers was highly variable and heterogeneous. These results were somewhat expected as research shows this pattern in other age-related chronic diseases such as hypertension and diabetes, two highly comorbid health conditions in patients with kidney failure. In the context of in-center hemodialysis, modest associations may not necessarily reflect the lack of precision of the questionnaires to assess adherence, but rather a limitation of the clinical biomarkers used as criterion measures. In this sense, direct measurement of potassium and phosphorus intake can be confounded by several clinical outcomes such as the prescribed dialysis dosage (e.g., number of treatments per week and minutes of each dialysis session) and type (e.g., conventional hemodialysis vs on-line hemodiafiltration), presence of co-morbid conditions (e.g., presence of diabetes mellitus), and/or prescribed medication (e.g., class of phosphate binders) [59, 63–66]. Still, most primary studies failed to consider the impact of these confounding factors in their analysis, which may contribute to heterogeneity and the small strength associations between biomarkers and self-reported adherence.

### Limitations

The primary studies only allowed conclusions to be drawn about the concurrent validity of self-report measures, leaving their predictive potential unexplored. The evidence of this systematic review with Hunter-Schmidt’s psychometric meta-analyses is also based on studies with different sample sizes, which makes it difficult to compare results. In addition, the results are based on studies with important methodological limitations such as the low control of potential confounders. Lastly, the individual meta-analyses provided evidence from a small number of studies with considerable heterogeneity; therefore, results should be interpreted with caution and viewed as preliminary.

## Conclusion

The question of how to optimize the measurement of adherence in hemodialysis emerges as a key issue that needs to be addressed, given the wide variability in non-adherence rates between studies. The results showed that the test scores of the ESRD-AQ and DDFQ have medium to large strength associations with IDWG, SPO, and SK values, indicating that these questionnaires have reasonable concurrent criterion validity to measure fluid control, adherence to dietary restrictions, and overall non-adherence in patients undergoing in-center hemodialysis. However, several small-strength and considerably heterogeneous associations were found; therefore, the decision to use one questionnaire over another must be made with caution and consider the characteristics of the sample and the objectives of the study. Given that direct and indirect methods have their advantages and disadvantages, the combination of adherence measures is recommended to accurately assess this complex and multidimensional outcome. Future studies are needed to develop and assess the reliability of composite measures to increase confidence in hemodialysis adherence outcomes.

## Supporting information

S1 Checklist PRISMA 2020 checklist. (PDF)

S1 File Protocol registered in PROSPERO. (PDF)

S1 Table Search strategy for all databases. (PDF)

S2 Table Critical appraisal of the primary studies. (PDF)

S3 Table Confounding factors in primary studies. (PDF)

S4 Table Summary of each self-report adherence measure analyzed in this systematic review. (PDF)
