# Supplementary material for: Establishing the criterion validity of self-report measures of adherence in hemodialysis through associations with clinical biomarkers: A systematic review and meta-analysis
Source: PLoS One. 2022 Oct 18;17(10):e0276163. doi: 10.1371/journal.pone.0276163 (PMC9578604; doi:10.1371/journal.pone.0276163)
Supplement: S1 File — (PDF) [file pone.0276163.s002.pdf]

To enable PROSPERO to focus on COVID-19 submissions, this registration record has undergone basic automated checks for eligibility and is published exactly as submitted. PROSPERO has never provided peer review, and usual checking by the PROSPERO team does not endorse content. Therefore, automatically published records should be treated as any other PROSPERO registration. Further detail is provided here.

Citation

Helena Sousa, Oscar Ribeiro, Elísio Costa, Alan Christensen, Daniela Figueiredo. Self-report measures and biomarkers of treatment (non)adherence in end-stage renal disease: A systematic review. PROSPERO 2021 CRD42021267550 Available from:
https://www.crd.york.ac.uk/prospero/display_record.php?ID=CRD42021267550

Review question

What is the criterion validity of self-report measures of adherence established through the association with clinical biomarkers likely to be affected by patients' adherence in end-stage renal disease?

Searches

Studies were identified by searching Scopus, Web of Science Core Collection, Current Contents Connect, Derwent Innovations Index, KCI-Korean Journal Database, Russian Science Citation Index, SciELO Citation Index, MEDLINE, CINAHL, and APA PsycINFO. The following keywords were used: dialysis OR hemodialysis OR haemodialysis OR end-stage renal disease OR end-stage kidney disease OR renal failure OR renal replacement therapy AND adherence OR compliance OR self-management OR self-care AND self-report OR questionnaire OR measure OR scale OR instrument AND albumin OR potassium OR phosphorus OR phosphate OR interdialytic weight OR IDWG OR Kt/V OR blood urea nitrogen OR calcium OR sodium OR creatinine OR biomarkers. These keywords were defined based on the inclusion criteria, by consulting other reviews related to treatment adherence in ESRD (Lambert et al., 2017) and the most recently published National Kidney Foundation's guidelines (Ikizler et al., 2020; Mallamaci et al., 2020; National Kidney Foundation, 2015; Lok et al., 2020). Limits were applied for English language and research with adults. Studies published prior to 2000 and grey literature (e.g., other reviews, protocols, practice guidelines, conference abstracts) were not considered. The reference lists of the identified studies and other reviews were hand searched to ensure that all important studies were included.

Types of study to be included

All study designs will be included, except for qualitative studies, reviews, editorials or case reports. For longitudinal or experimental studies with pre- and post-assessment points, data will be collected only for baseline results.

Condition or domain being studied

End-stage renal disease (ESRD).

Participants/population

Studies that assessed adults with ESRD (? 18 years old) undergoing hemodialysis will be included. Studies that focused only on other renal replacement therapies, such as peritoneal dialysis or kidney transplantation, will be excluded. This decision is based on the results of previous research, suggesting that hemodialysis is the most demanding renal replacement therapy with higher non-adherence rates (Lambert et al., 2017; Murali et al., 2019; Murali & Lonergan, 2020). No limits will be applied for time on dialysis.

Intervention(s), exposure(s)

Studies that exposed patients to valid and reliable self-report measures of treatment adherence in ESRD. Studies will be included if assessing one or more domains of treatment adherence (e.g., dietary, fluid, or medication adherence) (Murali et al., 2019). Self-made questionnaires, visual analogue scales, and/or measures without validation data in the context of ESRD will be excluded, as recommended by Stirrat and colleagues (2015).

Comparator(s)/control

Studies also assessing clinical biomarkers likely to be affected by treatment adherence in ESRD, according to the most recently published National Kidney Foundation’s guidelines (Ikizler et al., 2020; National Kidney Foundation, 2015; Lok et al., 2020). Only studies that report the results of the association between self-report measures and adherence biomarkers will be included.

Context

Not applicable

Main outcome(s)

The criterion validity of self-report measures of treatment adherence established through the association with clinical biomarkers likely to be affected by patients’ adherence is the primary outcome of interest (Berg & Arnsten 2006; Cohen and Swerdlik, 2018). This systematic review also aims to classify the most commonly used direct (i.e., biological endpoints or biomarkers) and indirect (i.e., self-report) measures of adherence outcomes in ESRD.

Measures of effect

The validity coefficient will be used as an effect measure (Cohen and Swerdlik, 2018). This refers to a correlation coefficient that provides a measure of the relationship between the test scores (i.e., self-report measures of treatment adherence) and scores of the other measure of interest (i.e., biomarkers). Typically, the Pearson correlation or Spearman Rho’s coefficients are used to determine the criterion validity between two measures (Cohen and Swerdlik, 2018).

Additional outcome(s)

Treatment adherence, defined as the extent to which a person’s behavior corresponds with agreed recommendations from a health care provider (WHO, 2003), is the secondary outcome. In patients undergoing hemodialysis, such behavior includes the adherence to dietary recommendations, fluid restrictions, medication intake, attending dialysis sessions without missing or shortening sessions, perform regular physical exercise, and care for the vascular access (Lok et al., 2020; Mallamaci et al., 2020; Murali et al., 2019; Murali & Lonergan, 2020).

Measures of effect

The validity coefficient will be used as an effect measure (Cohen and Swerdlik, 2018). This refers to a correlation coefficient that provides a measure of the relationship between the test scores (i.e., self-report measures of treatment adherence) and scores of the other measure of interest (i.e., biomarkers). Typically, the Pearson correlation or Spearman Rho’s coefficients are used to determine the criterion validity between two measures (Cohen and Swerdlik, 2018).

Data extraction (selection and coding)

The findings will be reported using the 2020 Preferred Reporting Items for Systematic Reviews and Meta-Analysis (PRISMA) statements (Page et al., 2021). One author will extract the studies from the databases and import them to Rayann, a software designed to facilitate the study selection in systematic reviews (https://www.rayyan.ai/). After manually removing duplicates, the unique studies will be selected over three steps: (i) studies will be labeled as included, excluded, or unclear, based on title and abstract; (ii) those studies labeled as unclear or as included will be retrieved; and (iii) the full text will be analyzed. Two authors

will independently perform the eligibility assessments. Discrepancies will be resolved by discussion and consensus, and by consulting with a third author. Data will be directly extracted from the eligible studies and organized into a Microsoft Excel Spreadsheet by one researcher and confirmed by another. The following data will be retrieved: studies´ characteristics (date, design and sample size), samples' characteristics (age, sex, country, and time on dialysis), self-report measures of treatment adherence and its results, biomarkers and its results, statistics used to explore the association between self-reported measures and biomarkers, and the major results for this association.

Risk of bias (quality) assessment

Critical appraisal will be performed using PRISMA recommendations while consulting the Joanna Briggs Institute (JBI) Critical Appraisal Checklists 2017, using the appropriate checklist for each study design. These checklists aim to assess the methodological quality of a study regarding the possibility of bias in its design, conduct and analysis. Two authors will conduct the appraisal and an inter-rater agreement will be calculated. Discrepancies will be resolved by discussion and consensus, and by consulting with a third author.

Strategy for data synthesis

Continuous variables will be expressed as means and standard deviations, and proportions will be expressed as percentages. A qualitative data synthesis will be conducted based on the consistency of the findings and their statistical significance set at a p value of <.05 (van der Berg, 2013).

Analysis of subgroups or subsets

Not applicable

Contact details for further information

Helena Sousa
helena.sousa@ua.pt

Organisational affiliation of the review

University of Aveiro

Review team members and their organisational affiliations

Miss Helena Sousa. University of Aveiro
Professor Oscar Ribeiro. University of Aveiro
Professor Elísio Costa. University of Porto
Professor Alan Christensen. East Carolina University
Professor Daniela Figueiredo. University of Aveiro

Type and method of review

Systematic review

Anticipated or actual start date

01 June 2021

Anticipated completion date

11 September 2021

Funding sources/sponsors

This work is financially supported by a PhD grant scholarship (reference number DFA/BD/4821/2020) attributed to Helena Sousa, financed by FCT (Fundação para a Ciência e Tecnologia) through FSE (Fundo Social Europeu).

Grant number(s)

State the funder, grant or award number and the date of award

DFA/BD/4821/2020

Conflicts of interest
No potential conflict of interest is reported by the authors.
None known

Language
English

Country
Portugal

Stage of review
Review Ongoing

Subject index terms status
Subject indexing assigned by CRD

Subject index terms
MeSH headings have not been applied to this record

Date of registration in PROSPERO
14 August 2021

Date of first submission
14 July 2021

Stage of review at time of this submission

Stage	Started	Completed
Preliminary searches	Yes	Yes
Piloting of the study selection process	Yes	Yes
Formal screening of search results against eligibility criteria	No	No
Data extraction	No	No
Risk of bias (quality) assessment	No	No
Data analysis	No	No

The record owner confirms that the information they have supplied for this submission is accurate and complete and they understand that deliberate provision of inaccurate information or omission of data may be construed as scientific misconduct.
The record owner confirms that they will update the status of the review when it is completed and will add publication details in due course.

Versions
14 August 2021
14 August 2021
