# Supplementary material for: Establishing the criterion validity of self-report measures of adherence in hemodialysis through associations with clinical biomarkers: A systematic review and meta-analysis
Source: PLoS One. 2022 Oct 18;17(10):e0276163. doi: 10.1371/journal.pone.0276163 (PMC9578604; doi:10.1371/journal.pone.0276163)
Supplement: S1 Table — (PDF) [file pone.0276163.s003.pdf]

S1 Table. Search strategy for all databases.

	Web of Science (all databases included)	SCOPUS	PsycInfo	CINHAL	MEDLINE/PubMed
**Search and keywords**	Title=(dialysis OR hemodialysis OR haemodialysis OR end-stage renal disease OR end-stage kidney disease OR renal failure OR renal replacement therapy) AND Topic=(adherence OR compliance OR self-management OR self-care) AND Topic=(self-report OR questionnaire OR measure OR scale OR instrument) AND Topic=(albumin OR potassium OR phosphorus OR phosphate OR interdialytic weight OR IDW OR IDWG OR Kt/V OR blood urea nitrogen OR calcium OR PCR OR sodium OR creatinine OR biomarkers)	Abstract= (dialysis OR hemodialysis OR haemodialysis OR "end-stage renal disease" OR "end-stage kidney disease" OR "renal failure" OR "renal replacement therapy") AND Abstract= (adherence OR compliance OR "self-management" OR "self-care") AND ALL=(self-report OR questionnaire OR measure OR scale OR instrument) AND ALL=(albumin OR potassium OR phosphorus OR phosphate OR interdialytic weight OR idw OR idwg OR kt/v OR blood urea nitrogen OR calcium OR pcr OR sodium OR creatinine OR biomarkers)	Title=(dialysis OR hemodialysis OR haemodialysis OR end-stage renal disease OR end-stage kidney disease OR renal failure OR renal replacement therapy) AND Full-text=(adherence OR compliance OR self-management OR self-care) AND Full-text=(self-report OR questionnaire OR measure OR scale OR instrument) AND Full-text=(albumin OR potassium OR phosphorus OR phosphate OR interdialytic weight OR IDW OR IDWG OR Kt/V OR blood urea nitrogen OR calcium OR PCR OR sodium OR creatinine OR biomarkers)	Title=(dialysis OR hemodialysis OR haemodialysis OR end-stage renal disease OR end-stage kidney disease OR renal failure OR renal replacement therapy) AND Full-text=(adherence OR compliance OR self-management OR self-care) AND Full-text=(self-report OR questionnaire OR measure OR scale OR instrument) AND Full-text=(albumin OR potassium OR phosphorus OR phosphate OR interdialytic weight OR IDW OR IDWG OR Kt/V OR blood urea nitrogen OR calcium OR PCR OR sodium OR creatinine OR biomarkers)	(((dialysis[Title/Abstract] OR hemodialysis[Title/Abstract] OR haemodialysis[Title/Abstract] OR end-stage renal disease[Title/Abstract] OR end-stage kidney disease[Title/Abstract] OR renal failure[Title/Abstract] OR renal replacement therapy[Title/Abstract]) AND (adherence[Title/Abstract] OR compliance[Title/Abstract] OR self-management[Title/Abstract] OR self-care[Title/Abstract])) AND (self-report[Title/Abstract] OR questionnaire[Title/Abstract] OR measure[Title/Abstract] OR scale[Title/Abstract] OR instrument[Title/Abstract])) AND (albumin[Title/Abstract] OR potassium[Title/Abstract] OR phosphorus[Title/Abstract] OR phosphate[Title/Abstract] OR interdialytic weight[Title/Abstract] OR IDW[Title/Abstract] OR IDWG[Title/Abstract] OR Kt/V[Title/Abstract] OR blood urea nitrogen[Title/Abstract] OR calcium[Title/Abstract] OR PCR[Title/Abstract] OR sodium[Title/Abstract] OR creatinine[Title/Abstract] OR biomarkers[Title/Abstract])

**Limits applied**	Exclude grey literature, journals related to children and adolescence or psychiatric conditions. English only.	Exclude all journals related to children and adolescence or psychiatric conditions. English only.	Exclude publications prior to 1990, studies with population under 18 years old, and qualitative studies. English only.	Exclude publications prior to 1990. English only.	Exclude publications prior to 1990. English only.
