# Supplementary material for: Establishing the criterion validity of self-report measures of adherence in hemodialysis through associations with clinical biomarkers: A systematic review and meta-analysis
Source: PLoS One. 2022 Oct 18;17(10):e0276163. doi: 10.1371/journal.pone.0276163 (PMC9578604; doi:10.1371/journal.pone.0276163)
Supplement: S2 Table — (PDF) [file pone.0276163.s004.pdf]

S2 Table. Critical appraisal of the primary studies.

Cross-sectional studies	Q1	Q2	Q3	Q4	Q5	Q6	Q7	Q8	Appraisal
**Ahrari et al., 2014**	Yes	Yes	Yes	Yes	NA	NA	Yes	Yes	Included
**Amado et al., 2015**	Yes	Yes	Yes	Yes	NA	NA	Yes	Yes	Included
**Antony et al., 2020**	Yes	Yes	Yes	Yes	NA	NA	Yes	Yes	Included
**Anuja et al., 2020**	Yes	Yes	Yes	Yes	No	No	Yes	Yes	Included
**Chan et al., 2012**	Yes	Yes	Yes	Yes	Yes	Yes	Yes	Yes	Included
**Chao et al., 2016**	Yes	Yes	Yes	Yes	NA	NA	Unclear	Yes	Included
**Chen et al., 2021**	Yes	Yes	Yes	Yes	Yes	Yes	Yes	Yes	Included
**Daniels et al., 2018**	Yes	Yes	Yes	Yes	No	No	Unclear	Yes	Included
**Efe et al., 2015**	Yes	Yes	Yes	Yes	NA	NA	Yes	Yes	Included
**Fincham et al., 2008**	Yes	Yes	Yes	Yes	NA	NA	Yes	Yes	Included
**Ghimire et al., 2016**	Yes	Yes	Yes	Yes	NA	NA	Yes	Yes	Included
**Joson et al., 2016**	Yes	Yes	Yes	Yes	NA	NA	Yes	Yes	Included
**Kara et al., 2007**	Yes	Yes	Yes	Yes	NA	NA	Unclear	Yes	Included
**Katalinic et al., 2017**	Yes	Yes	Yes	Yes	NA	NA	Yes	Yes	Included
**Kauric-Klein et al., 2013**	Yes	Yes	Yes	Yes	NA	NA	Yes	Yes	Included
**Khalil et al., 2013**	Yes	Yes	Yes	Yes	NA	NA	Yes	Yes	Included
**Kim et al., 2010**	Yes	Yes	Yes	Yes	Yes	Yes	Yes	Yes	Included
**Kugler et al., 2005**	Yes	Yes	Yes	Yes	NA	NA	Unclear	Yes	Included
**Lim et al., 2020**	Yes	Yes	Yes	Yes	NA	NA	Yes	Yes	Included
**Mellon et al., 2013**	Yes	Yes	Yes	Yes	NA	NA	Yes	Yes	Included
**Mollaoğlu et al., 2015**	Yes	Yes	Yes	Yes	Yes	Yes	Yes	Yes	Included
**Naalweh et al., 2017**	Yes	Yes	Yes	Yes	NA	NA	Yes	Yes	Included

**Ok et al., 2019**	Yes	Yes	Yes	Yes	NA	NA	Yes	Yes	Included
**Poveda et al., 2016**	Yes	Yes	Yes	Yes	NA	NA	Yes	Yes	Included
**Umeukeje et al., 2015**	Yes	Yes	Yes	Yes	Yes	Yes	Yes	Yes	Included
**Umeukeje et al., 2016**	Yes	Yes	Yes	Yes	Yes	Yes	Yes	Yes	Included
**Vlaminck et al., 2001**	Yes	Yes	Yes	Yes	NA	NA	Yes	Yes	Included
**Wileman et al., 2014**	Yes	Yes	Yes	Yes	NA	NA	Yes	Yes	Included
**Wileman et al., 2011**	Yes	Yes	Yes	Yes	Yes	Yes	Yes	Yes	Included

Q = question number; NA = Not Applicable.
