# Supplementary material for: Establishing the criterion validity of self-report measures of adherence in hemodialysis through associations with clinical biomarkers: A systematic review and meta-analysis
Source: PLoS One. 2022 Oct 18;17(10):e0276163. doi: 10.1371/journal.pone.0276163 (PMC9578604; doi:10.1371/journal.pone.0276163)
Supplement: S3 Table — (PDF) [file pone.0276163.s005.pdf]

**S3 Table. Confounding factors in primary studies.**

| Reference | Residual renal function | Type of HD (conventional or on-line HDF) | Nº of HD sessions per week | ESRD etiology | Type of vascular access | When/how were biomarkers collected? |
|---|---|---|---|---|---|---|
| Ahrari et al., 2014 | NR | NR | NR | NR | NR | Pre-dialysis |
| Amado et al., 2015 | NR | On-line HDF | Thrice in a week/ 4h per session | NR | CVC: 22%<br>AVF: 88% | Pre-dialysis |
| Antony et al., 2020 | NR | NR | Twice in a week (62% of patients) | NR | NR | Pre-dialysis and in the same week of the questionnaire administration |
| Anuja et al., 2020 | NR | NR | NR | NR | NR | Pre-dialysis |
| Chan et al., 2012 | NR | NR | NR | Diabetes: 26%<br>Glomerulonephritis: 8.5% | NR | Pre-dialysis and the average of the last 3 months before the questionnaire administration |
| Chao et al., 2016 | NR | NR | NR | Diabetes: 40% | NR | NR |
| Chen et al., 2021 | NR | NR | Thrice in a week | NR | NR | Pre-dialysis and post-dialysis weight data from 3 consecutive dialysis sessions close to questionnaire administration |
| Daniels et al., 2018 | NR | NR | NR | NR | NR | NR |
| Efe et al. 2015 | NR | NR | NR | NR | NR | Pre-dialysis and in the same week of the questionnaire administration |
| Fincham et al., 2008 | NR | NR | NR | NR | NR | Pre-dialysis and the average of the last 3 months before the questionnaire administration |
| Ghimire et al., 2016 | NR | NR | NR | NR | NR | Pre-dialysis and in the same week of the questionnaire administration |
| Joson et al., 2016 | NR | NR | NR | NR | NR | Pre-dialysis and the average of the last 3 months before the questionnaire administration |
| Kara et al., 2007 | NR | NR | NR | NR | NR | NR |
| Katalinić et al., 2017 | *M* = 183.48 ml/day (0-4000) – negative correlation with medication adherence (r = -.119, $p$<.05), measured with the MMAS | NR | NR | NR | NR | Pre-dialysis and the average of the last 3 months before the questionnaire administration |

| | | | | | | |
|---|---|---|---|---|---|---|
| Kauric-Klein et al., 2013 | NR | NR | NR | NR | NR | Pre-dialysis and the last BPs were averaged |
| Khalil et al., 2013 | 217 ml/day – less residual function is associated with lower adherence to diet ($r = -.21$, $p = .02$) and to fluid ($r = -.19$, $p = .01$) restrictions, measured by the DDFQ. | NR | NR | NR | NR | Pre-dialysis and the average of the last 3 months before the questionnaire administration |
| Kim et al., 2010 | NR | NR | 3h per session (25.8%) | Diabetes: 43.0% Hypertension: 26.5% | NR | Average IDGW for the 12 sessions during the four weeks preceding the day the questionnaire was completed; pre-dialysis biochemical markers obtained on the day closest to the questionnaire administration |
| Kugler et al., 2005 | NR | NR | NR | NR | NR | NR |
| Lim et al., 2020 | NR | NR | NR | NR | NR | Pre-dialysis and the average of the last 3 months before the questionnaire administration |
| Mellon et al., 2013 | NR | NR | NR | NR | NR | Average IDWG for the 6 sessions during the 2 weeks preceding the day the questionnaire was completed; pre-dialysis biochemical markers were obtained by averaging levels over 3 months. |
| Mollaoğlu et al., 2015 | NR | NR | NR | NR | NR | Average IDGW was obtained by averaging all measurements over 3 months before questionnaire administration |
| Naalweh et al., 2017 | NR | NR | Twice in a week/3h per session | NR | NR | Average of the last 3 measurements in the past month |
| Ok et al., 2019 | NR | NR | Thrice in a week/ 3-4h per session | NR | NR | Average IDWG for the 6 sessions during the 2 weeks preceding the day the questionnaire was completed; pre-dialysis biochemical markers were measured in the last month before the questionnaire administration |
| Poveda et al., 2016 | NR | On-line HDF | Thrice in a week/ 4h per session | Diabetes: 38.4% Hypertension: 62.2% | NR | Pre-dialysis and in the same week of the questionnaire administration |
| Umeukeje et al., 2015 | NR | NR | NR | NR | NR | The most recent monthly pre-dialysis serum phosphorus level closest to the date of the questionnaire application |

| | | | | | | |
|---|---|---|---|---|---|---|
| Umeukeje et al., 2016 | NR | NR | NR | NR | NR | The most recent monthly pre-dialysis serum phosphorus level closest to the date of the questionnaire application |
| Vlaminck et al., 2001 | NR | NR | NR | NR | NR | IDWG increase in weight in kg after a weekend; pre-dialysis biochemical markers were the average of the last 3 measures |
| Wileman et al., 2014 | Patients were included only if the residual renal function was below 1 ml/min. KRU did not significantly predict adherence to medication. | NR | NR | Diabetes: 14% Hypertension: 11% Glomerulonephritis: 8% | NR | Pre-dialysis and the average of the last 3 months before the questionnaire administration |
| Wileman et al., 2011 | Most patients had a residual renal function of < 1 ml/min. KRU was not a significant predictor of medication adherence (*beta* = .01, *p* = .92) | NR | NR | NR | NR | Pre-dialysis and the average of the last 3 months before the questionnaire administration |

NR = Not Reported.
