# Supplementary material for: Establishing the criterion validity of self-report measures of adherence in hemodialysis through associations with clinical biomarkers: A systematic review and meta-analysis
Source: PLoS One. 2022 Oct 18;17(10):e0276163. doi: 10.1371/journal.pone.0276163 (PMC9578604; doi:10.1371/journal.pone.0276163)
Supplement: S4 Table — (PDF) [file pone.0276163.s006.pdf]

S4 Table. Summary of each self-report adherence measure analyzed in this study.

**Self-report measures of treatment adherence (author)**	**The End-Stage Renal Disease – Adherence Questionnaire (ESRD-AQ)**	**The Dialysis Diet and Fluid Non-Adherence Questionnaire (DDFQ)**	**The Renal Adherence Behavior Questionnaire (RABQ)**	**Fluid Adherence Subscale (from the Hemodialysis Patients Therapy Adherence Scale)**	**The Morisky Medication Adherence Scale (MMAS)**	**The Medication Adherence Rating Scale (MARS)**
**Original validation**	Kim Y, Evangelista LS, Phillips LR, Pavlish C, Kopple JD. The End-Stage Renal Disease Adherence Questionnaire (ESRD-AQ): testing the psychometric properties in patients receiving in-center hemodialysis. *Nephrol Nurs J*. 2010;37(4):377-393.	Vlaminck H, Maes B, Jacobs A, Reyntjens S, Evers G. The dialysis diet and fluid non-adherence questionnaire: validity testing of a self-report instrument for clinical practice. *J Clin Nurs*. 2001;10(5):707-715.	Rushe H, McGee HM. Assessing adherence to dietary recommendations for hemodialysis patients: The Renal Adherence Attitudes Questionnaire (RAAQ) and the Renal Adherence Behavior Questionnaire (RABQ). *J Psychosom Res*. 1998;45(2):149-157.	Zhang Y. The development of a medical treatment adherence scale for end-stage renal disease clients with maintenance hemodialysis. Masters' Thesis, Central South University. 2012.	Morisky DE, Green LW, Levine DM. Concurrent and predictive validity of a self-reported measure of medication adherence. *Med Care.* 1986; 24:67–74. Morisky DE, Ang A, Krousel-Wood M, Ward HJ. Predictive validity of a medication adherence measure in an outpatient setting. J *Clin Hypertens* 2008;10(5):348-354.	Thompson K, Kulkarni J, Sergejew AA. Reliability and validity of a new Medication Adherence Rating Scale (MARS) for the psychoses. *Schizophr Res*. 2000;42(3):241-247.
**Type of adherence measured**	Hemodialysis attendance, medication intake, fluid	Fluid restrictions, and diet recommendations	Adherence to restrictions on potassium, sodium,	Fluid restrictions	Medication intake	Medication intake

	restrictions, and diet recommendations.		fluids, and medication intake.			
**Assessment time-point**	During the *last month.*	During the *14 days.*	Not applicable.	NR	Not applicable.	During the past week.
**Number of items**	46 items, divided into 5 sections: (i) general information the patients' condition (items 1 -5), (ii) hemodialysis attendance (items 6-19), (iii) medication intake (items 20-28), (iv) fluid restriction (items 29-38), and (v) dietary restriction (items 39-46).	4-item: two for the measurement of non-adherence behavior with fluids and two for the assessment of diet non-adherence (frequency and degree).	25 items, divided into 5 subscales: (i) adherence to fluid restrictions (11 items), (ii) adherence to potassium/phosphate medication (5 items), (iii) self-care (2 items), (iv) adherence in times of particular difficulty (5 items), (v) and adherence to sodium restrictions (2 items).	This subscale has six items and uses a 5-point scale	MMAS has two versions: MMAS-4 (4 items, including elements of forgetfulness and symptom severity) and MMAS-8 (8 items that add other situational and emotional aspects of medication adherence).	10 items
**Type of items**	Likert, multiple-choice, and "yes/no" answers.	5-point Likert scale from "no" to "very severe".	5-point Likert scale from ''never'' to ''always.	5-point Likert from "never did" to "totally did".	Response categories are "yes/no" for each item and a 5-point Likert response for the last item.	"Yes/no" answers.
**Scoring**	Hemodialysis attendance (0-300, item 14, and 0-200 item 17), medication intake attendance (0-200, item 26), fluid restrictions (0-200, item 31), and diet	Frequency is measured as the number of days; degree is measured from 0 to 4, with higher scores indicating greater non-adherence.	Higher scores indicate greater adherence.	NR	Higher scores indicate greater adherence.	Higher scores indicate greater adherence.

	recommendations (0-200, item 46). Overall nonadherence is calculated by summing up items 14, 17, 18, 26, 31, and 36. Higher scores indicate greater non-adherence.					
**Psychometric qualities**	High validity and reliability; the item-level content validities for the 46 items ranged between 0.86 and 1.00; ICCs range from 0.83 to 1.00.	Moderate construct validity (τ = 0.495-0.678).	Moderate to strong internal consistency (α = 0.56-0.80) and test-retest reliability (r = 0.69-0.91).	Good internal consistency in the development study (α = 0.89).	Strong internal consistency (α = 0.83); good convergent validity between MMAS-4 and MMAS-8 (r = 0.64)	Moderate to strong internal consistency (α = 0.75) and test-retest reliability (χ2= 0.72).
